# Argon release, crystallization, morphological and optical changes of ion-beam sputtered Ta_2_O_5_ thin films during thermal treatments

**DOI:** 10.1016/j.heliyon.2025.e42009

**Published:** 2025-01-16

**Authors:** A. Paolone, M. Bazzan, G. Favaro, F. Borondics, G. Nemeth, F. Capitani, S. Swaraj, R. Belkhou, R. Flammini, J. Teillon, C. Michel, D. Hofman, M. Granata

**Affiliations:** a Istituto dei Sistemi Complessi, Consiglio Nazionale delle Ricerche, Piazzale A. Moro 5, 00185, Roma, Italy; b Sezione di Roma, Istituto Nazionale di Fisica Nucleare, Piazzale A. Moro 5, 00185, Roma, Italy; c Dipartimento di Fisica e Astronomia, Università di Padova, Via F. Marzolo 8, 35131, Padova, Italy; d Sezione di Padova, Istituto Nazionale di Fisica Nucleare, Via F. Marzolo 8, 35131, Padova, Italy; e SOLEIL Synchrotron, L'Orme des Merisiers, RD 128, 91190, Saint Aubin, France; f Istituto di Struttura della Materia, Consiglio Nazionale delle Ricerche, Area di Ricerca di Tor Vergata, via del Fosso del Cavaliere 100, 00133, Roma, Italy; g Laboratoire des Matériaux Avancés – IP2I, CNRS, Université de Lyon, 69622, Villeurbanne, France

**Keywords:** Amorphous materials, Gravitational wave detectors, Tantala, Thermal treatments, Crystallization, Optical properties

## Abstract

Mass spectrometry measurements at high temperatures on amorphous Ta_2_O_5_, produced by Ion Beam Sputtering (IBS) and widely used for the high reflective coatings of the mirrors of gravitational-wave detectors, detect a release of argon around 1000 K. This process is thermally activated and the corresponding activation energy is 410 ± 60 kJ/mol. X-Ray Diffraction (XRD) measurements indicate that in the same range of temperature the crystallization of tantala takes place. Atomic Force Microscopy (AFM) experiments detect a reduction of the surface roughness in the crystalline phase; domains with typical dimensions of the order of few microns appear in the crystal. While the infrared reflectance and absorption properties measured by scanning near-field optical microscopy (SNOM) are extremely homogeneous in the amorphous state, they become dependent on the crystal domain at high temperatures. Moreover, an absorption band around 960 cm^−1^ appears only in samples heated in vacuum and not in specimen thermally treated in air.

Photoemission electron microscopy (PEEM) experiments support the idea that this infrared absorption band is due to the formation of non-homogeneous and non-stoichiometric oxygen domains.

## 1 Introduction

High precision cavities are essential elements of interferometric gravitational-wave detectors [1], optomechanical resonators [2], and frequency standards [3]. High reflective coatings are needed in all these applications; they are usually Bragg reflectors composed of a number of alternating layers of materials with low and high refractive index. At present the high-reflective coatings of the mirrors of the Advanced LIGO [4], Advanced Virgo [5], and KAGRA [6] gravitational-wave detectors are optimized stacks [7] of amorphous mixtures of titanium dioxide and tantalum pentoxide (Ta_2_O_5_, also known as tantala, with a high refractive index n_H_ ≈ 2.05) and amorphous silicon dioxide (SiO_2_, silica, with a low refractive index n_L_ ≈ 1.44) produced by the Laboratoire des Matériaux Avancés (LMA) by means of ion-beam sputtering (IBS) [[8], [9], [10]]. Despite its low overall thickness, Ta_2_O_5_ is the main source of optical absorption and mechanical loss of Advanced LIGO and Advanced Virgo mirrors [10]. To minimize such drawbacks some strategies were exploited, such as adding a significant amount of titanium dioxide (TiO_2_, titania) to the tantala high-index layers [[10], [11], [12], [13]] or performing high temperature thermal treatments [10,12,13].

In the last few years there has been also a large scientific effort to find different materials to replace Ta_2_O_5_ as the high refractive index layers [14]. Some of these new materials were also considered for gravitational-wave detectors working at cryogenic temperatures, such as KAGRA or Einstein Telescope. Among the proposed replacements for Ta_2_O_5_, one can cite other amorphous oxides such as Nb_2_O_5_ [15] and TiO_2_ [16] or mixtures of oxides (such as TiO_2_:Nb_2_O_5_ [15], TiO_2_:GeO_2_ [17], Ta_2_O_5_:SiO_2_ [18], or TiO_2_:SiO_2_ [19]); moreover, MgF_2_ [20] and AlF_3_ [21] attracted some attention, as well as amorphous SiH_x_ [22], SiC [23], SiNx [14] and SiNxH_y_ [24,25]. At present Ti:GeO_2_ displays lower mechanical loss than Ti:Ta_2_O_5_, while Ti:SiO_2_ has comparable optical absorption. At the same time, we are witnessing a renaissance of the investigation of this material from a fundamental point of view to improve its performances in applications.

Some directions of active research on Ta_2_O_5_ comprise the investigation of crystallization, the presence of argon as residue of the synthesis by Ion-Beam Sputtering (IBS) [[26], [27], [28]], the microstructure of the amorphous samples and the changes induced by thermal treatments [[28], [29], [30], [31], [32]], the presence of different impurities, such as hydrogen [33], a correlation between internal friction and Urbach energy [34].

The microstructure of amorphous Ta_2_O_5_ and its evolution with temperature has been a very active field. In fact Short Range Order (SRO) of Ta_2_O_5_ was measured starting from electron diffraction data [29] and SRO was found to slightly increase after thermal treatments. Extended X-ray absorption fine structure (EXAFS) measurements detected very weak differences between samples heated at 573 and 873 K [29]. The comparisons with the published crystalline structures showed that amorphous tantala is not a simple distortion of a crystalline structure and Ta atoms in amorphous tantala have fewer neighboring oxygen atoms than those in crystalline tantala [29]. More recently X-Ray pair distribution function measurements suggested that the arrangement of Ta atoms in amorphous Ta_2_O_5_ strongly resembles that of the high-pressure Z -Ta_2_O_5_ polymorph, whereas the topological properties of O atoms resemble that observed in the δ -Ta_2_O_5_ polymorph [32].

A special attention in the study of amorphous tantala has been devoted to the presence of argon retained in the structure following the synthesis by the IBS technique, as it is based on the sputtering from Ar atoms. A percentage ranging from 0.5 to 3 % of Ar is left in the amorphous structures [26,27,35]. The major part of Ar can be released from tantala upon thermal treatment with a maximum of the effusion around 573 K [26]. However, a very tiny quantity of argon was reported to be still present in the material and can coagulate forming bubbles at 873 K with typical dimensions of 22 Å [28]. The problem of the formation of bubbles is relevant from the application point of view, as they can disturb the optical transmission and/or the mechanical dissipation.

In the present paper, we will continue the investigation of amorphous Ta_2_O_5_ after thermal treatments started in Ref. [26], extending the temperature range up to 1123 K. We will focus on the desorption of Ar, revealing a new desorption process occurring in the same temperature range of crystallization. The morphologic and optical changes induced by crystallization will be studied. Finally a comparison between thermal treatment in vacuum and in air will be reported.

## 2 Materials and methods

All the investigated samples were cut from an amorphous Ta_2_O_5_ film, 103 nm thick, deposited on a crystalline Si wafer. The deposition was carried out by means of IBS in a VEECO Spector system. The ion beam used to promote the sputtering of a Ta metallic sample was composed of Ar ions accelerated and subsequently neutralized by means of an electron source. Argon was used in the ion-beam source while oxygen was fed into the chamber, reaching a total residual pressure of the order of 10^−4^ mbar in the chamber. The substrate was heated around 373 K; the ion energy and current were of the order of 1 keV and 0.5 A, respectively.

Thermal desorption spectroscopy (TDS) measurements were afterward performed ex-situ using an MTI GSL100 vacuum furnace equipped with a quartz tube, connected to a Pfeiffer Vacuum QMS200 mass spectrometer. The base pressure inside the tube was of the order of 10^−7^ mbar. Measurements were conducted with a heating temperature rate ranging between 1 and 10 K/min, between room temperature and 1123 K.

The optical system of a Raman DXR2 Thermo Fisher micro-spectrometer was used to acquire optical images of the pristine and thermally treated samples using an Olympus Mplan50X/0.75NA objective.

Ex-situ XRD experiments were conducted using a Philips MRD diffractometer working with the Cu K-α line produced by a tube operating at 40 kV and 40 mA. The monochromatization and collimation of the probing beam were achieved through the use of a parabolic Göbel mirror and a metallic slit. A proportional counter Xe detector equipped with a parallel plate collimator and a Soller slit was used to receive the diffracted radiation.

s-SNOM measurements were performed by means of an Attocube/NeaSpec NeaScope SNOM/TERS system in tapping mode. Simultaneous AFM and near field infrared (IR) images were obtained using quantum cascade lasers (QCL) at a fixed frequency. Typically, images were acquired with a spatial resolution of 50 nm on 10 μm × 10 μm areas in duplicate or triplicate to check for reproducibility. Additionally, broadband IR spectra were recorded in a few points of each sample in the range between 600 and 1600 cm^−1^ exploiting synchrotron radiation with a spectral resolution of about 3 cm^−1^.

X-ray PhotoEmission Electron Microscopy (PEEM) measurements were performed [36] by means of a 90° LEEM-PEEM manufactured by Elmitec GmbH (Germany). The X-PEEM instrument is equipped with a photoelectron energy analyzer to allow local X-ray photoelectron spectroscopy (XPS) measurements. The sample image is formed on a channel plate equipped with a CMOS Hamamatsu camera. A field of view of 25 μm was used. X-ray excitation energies of 200 eV for Tantalum 4f and 600 eV for Oxygen 1s core levels were employed to optimize the corresponding cross sections. Before analysis the data were normalized to the background image representing the variations and defects of the camera. The data were processed using the Fiji software.

## 3 Results and discussion

**Fig. 1:**
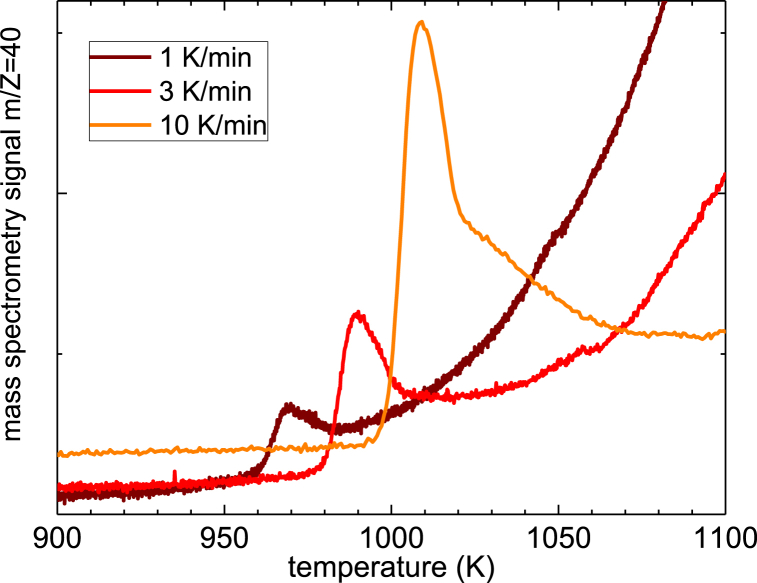
The argon effusion peak measured by TDS at different heating rates.

As reported in Ref. [26], a first massive release of argon from the Ta_2_O_5_ sample was revealed around 573 K; however, those measurements did not extend above 873 K. In the present paper TDS experiments were extended above 873 K up to 1123 K. Fig. 1 reports the Ar signal (m/Z = 40) detected for samples heated with a temperature rate of 1, 3 and 10 K/min. One can note a well-defined peak superimposed on a background; the latter is due to the inevitable increase of the pressure in the vacuum oven at high temperatures. By an integration of the peak, after removal of the background, using a calibration with different samples releasing Ar, we obtained that the quantity of Ar released during this high temperature process is about 3 parts over 10000 (in wt), which should be compared to the 3 parts over 1000 (in wt) present in the pristine sample, most of which is released around 573 K [26]. We measured two additional coatings with thickness of 4.5 and 953 nm and the area of the peak around 973 K does not display clear changes with the film thickness, differently from the case of the effusion argon peak observed around 573 K. Therefore, we proposed that while the effusion corresponding to the peak at 573 K was due to the release of argon from the bulk the Ta_2_O_5_ film [26], the peak at the highest temperature is due to the release of Ar from a well localized region of the film. Previous XPS measurements did not observe the presence of Ar on the external surface of Ta_2_O_5_ after thermal treatments at 873 K; it is highly plausible that this new peak is due to the release of Argon from the interphase between the Ta_2_O_5_ coating and the Si substrate.

The effusion peak around 1000 K becomes more intense and is shifted towards higher temperatures as the heating rate increases, moving from about 968 K for ΔT/dt = 1 K/min to about 1013 K for ΔT/dt = 10 K/min. The increase of the intensity is expected as the area of the peak (as a function of time) is proportional to the quantity of released Ar. As the releasing time is shortened at higher temperature rate, the peak intensity must increase, as visible in Fig. 1. The peak shift with the heating rate suggests that the release of Argon is a thermally activated process, which needs a given activation energy to occur, E_a_. Thermally activated processes are quite common and Kissinger developed a procedure to estimate the activation energy in physical or chemical processes from data obtained at several non-isothermal tests conducted at constant heating rates (constant during each test, different among tests) [37].

**Fig. 2:**
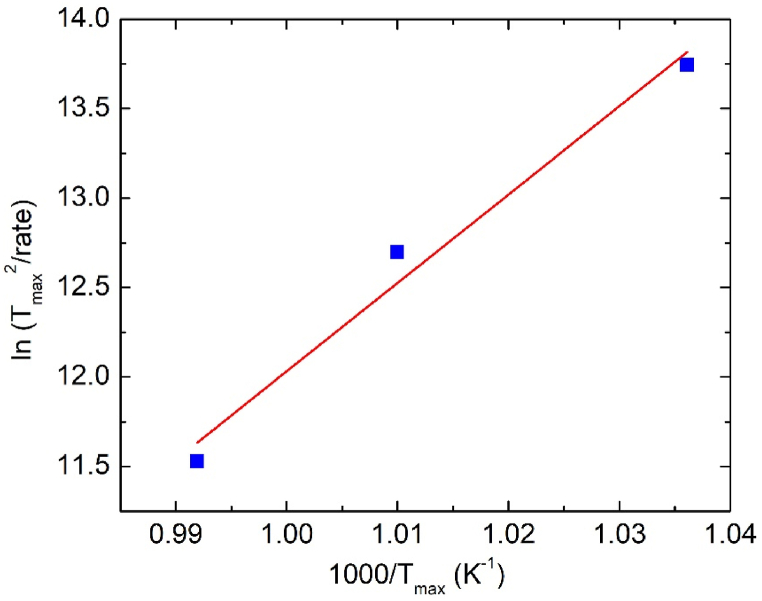
Kissinger plot of the Ar effusion peak centered around 1000 K and best fit line.

The energy barrier of the process, E_a_, can be calculated by means of a Kissinger plot, that relates the logarithm of the ratio between the square of the temperature of the maximum of the peak, T_max_, and the heating rate, φ, vs. the inverse of the peak maximum (see Fig. 2):

d(ln(φTmax2))d(1Tmax)=−EaR

where R is the gas constant. Fig. 2 shows that the data points are aligned along a straight line and the derived activation energy of the thermally activated process is 410 ± 60 kJ/mol.

One can note that the range of temperature of the Ar effusion peak corresponds to that of the crystallization of amorphous Ta_2_O_5_ thin films. Moreover, the value of activation energy of the effusion peak is of the same order of magnitude of that detected by differential scanning calorimetry during the crystallization of different amorphous or glassy materials [38]. Of course, the peak we detect in the present case is not directly related to crystallization, but the correspondence with the temperature range and with the energy values reported for the crystallization of similar materials suggests that the high temperature release of argon may take place concomitantly with the crystallization of amorphous tantala. It can be noted that the release of gases during the crystallization process of amorphous Ta_2_O_5_ was observed for nanotubes prepared by anodization in a sulfuric-acid-based solution, that released oxygen and sulfur when crystallization occurs [39].

**Fig. 3:**
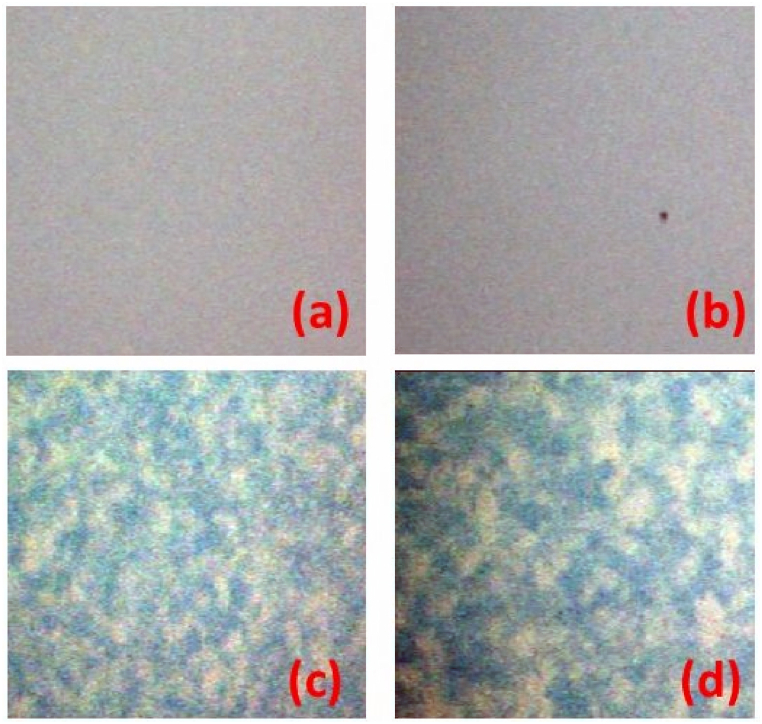
Optical microscopy images of the surfaces (40 × 40 μm^2^) of the four Ta_2_O_5_ samples: pristine (a) and after thermal treatments in vacuum at 988 K (b), at 1023 K (c) and at 1123 K (d).

The comparison of the optical microscopy images of the pristine and heated samples corroborates the hypothesis of crystallization in the same temperature range (see Fig. 3). Indeed, while the pristine Ta_2_O_5_ sample (Fig. 3a) and the sample heated in vacuum at 988 K (Fig. 3b) show a smooth surface, the samples heated at 1023 (Fig. 3c) and 1123 K (Fig. 3d) display a surface with clear domains having a typical linear size of the order of a few μm.

**Fig. 4:**
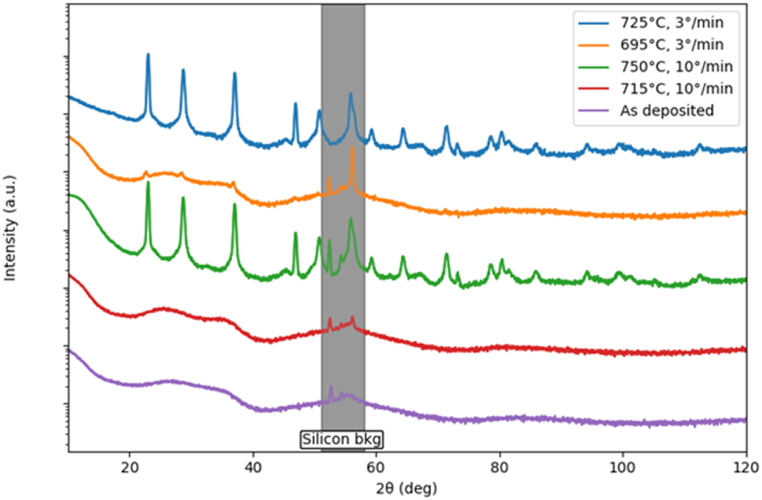
XRD diffraction patterns of pristine Ta_2_O_5_ (purple line) and after thermal treatments at 968 (orange) and 998 K (blue) with a heating temperature rate of 3 K/min or at 988 (red) and 1023 K (green) with a rate of 10 K/min. The shadowed region indicates the angular range in which the background coming from the Si wafer is visible. The curves have been multiplied by an arbitrary factor to make them easily visible on the same graph.

XRD measurements were conducted on the pristine and heated samples to ascertain the crystallization process. Fig. 4 reports the XRD diffraction patterns of five samples: the pristine one and two couples of samples annealed with different heating rates (3 and 10 K/min) just below and above the Ar effusion peak measured with the corresponding heating rate (see Fig. 1). The curves have been multiplied by an arbitrary factor to make them easily visible on the same graph. The as-deposited Ta_2_O_5_ shows only a smooth background due to the scattering from the amorphous matrix. The two samples heated below the Ar effusion peak (at 968 K with a heating rate of 3 K/min and at 988 K with a heating rate of 10 K/min) display features similar to the pristine sample. On the contrary, the two samples heated above the Ar effusion peak (at 998 K with a heating rate of 3 K/min and at 1023 K with a heating rate of 10 K/min) show clear diffraction peaks, definitively proving the crystallization of the amorphous structure. The diffraction pattern is coherent with other reports present in the literature [32,40,41] and can be attributed to crystalline tantala. The crystallization temperature of the presently investigated samples is consistent with that found for Ta_2_O_5_ [42] prepared by radio-frequency sputtering, while it is lower than that of highly doped Ta_2_O_5_ nanotubes obtained by anodization in a sulfuric-acid-based solution [39].

The average size τ of the coherent diffraction domains can be estimated by the Scherrer's equation:

$$\Delta =\frac{K\lambda }{\tau \;\cos\;\theta }$$

where Δ is the angular width of the diffraction peak corrected for the instrumental broadening which we quantified with the help of a LaB_6_ standard, K is a geometric factor equal to 0.9 in the case of spherical grains as it was assumed here for sake of simplicity, λ=1.54056 Å is the X-ray wavelength and θ is the Bragg angle. Our results point towards a grain size in the range of 50–100 nm for all the observed samples. This quantity is obviously much smaller than the grain sizes observed in Fig. 3 but the discrepancy can be easily explained considering that XRD can probe only the size of coherent diffraction domains which, in case of a defective structure, may be significantly smaller than the volume of a single crystalline grain.

**Fig. 5:**
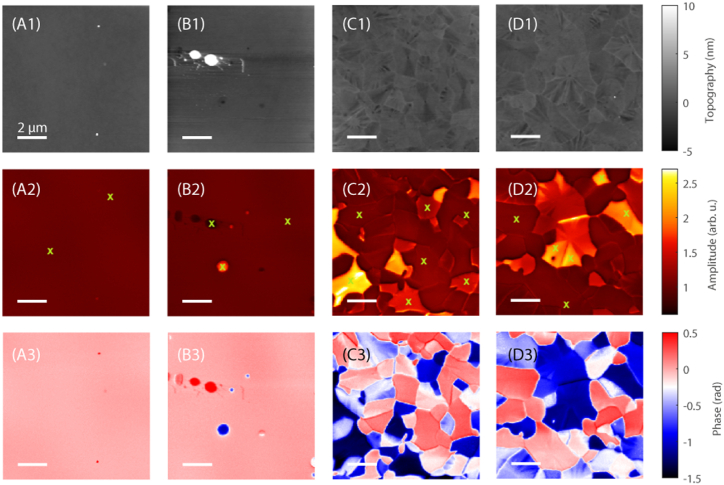
AFM topography (row 1), and near-field IR signals O3A (row 2) and O3P (row 3) at 959 cm^−1^ of the four Ta_2_O_5_ samples: pristine (column A) and after thermal treatments in vacuum at 988 K (column B), at 1023 K (column C) and at 1123 K (column D). The intensity scale span is equal to that reported for the pristine specimen in all samples. Little “x” mark the places where the spectra of Fig. 6 were collected.

**Fig. 6:**
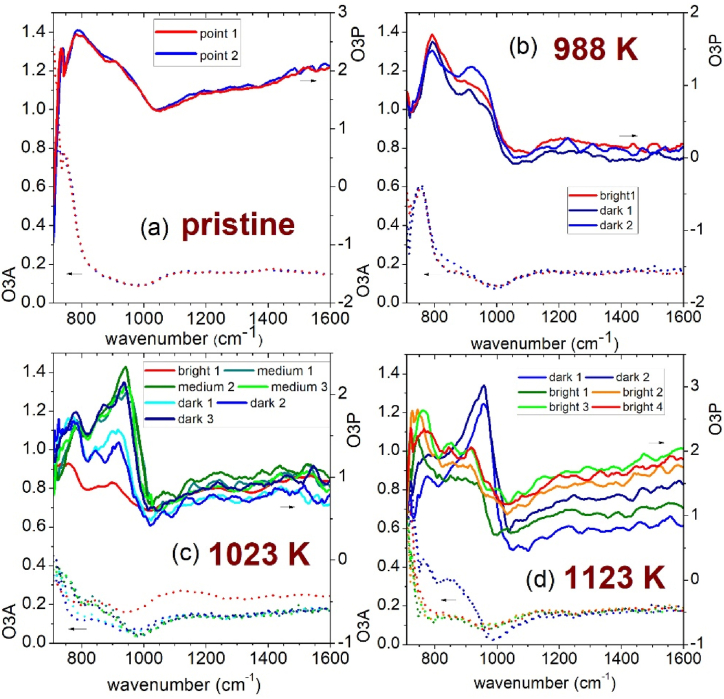
Broadband IR absorption spectra of the four Ta_2_O_5_ samples in different brightness point: pristine (a) and after thermal treatments in vacuum at 988 K (b), at 1023 K (c) and at 1123 K (d).

To evaluate possible changes in the surface morphology and in the optical properties of the films after thermal treatments, SNOM measurements were performed at the SMIS beamline of the Soleil Synchrotron. Fig. 5 reports the AFM topography (row 1) and the third-harmonic optical amplitude (O3A, row 2) and phase (O3P, row 3) of the IR near-field signals measured at 959 cm^−1^ for four Ta_2_O_5_ samples heated in vacuum, as in the mass spectrometry experiments. All values were normalized to the average signal level in the image and the color scale is set identical for all images to provide straight comparison. The specific value of the frequency was chosen in view of the peak that develops after thermal treatments (see subsequent Fig. 6c and d). The pristine sample displays a uniform surface (Fig. 5 Column A) with a root mean square roughness of 2.5 nm. The O3A and O3P signals, which usually are correlated to the reflectivity and absorbance of the samples, are almost constant, pointing out the uniformity of the coatings also from an optical point of view. On the contrary some circular structures are visible in the sample heated at 988 K in vacuum (Fig. 5 Column B); the mean roughness increases to 5.8 nm. At this temperature the sample is still amorphous, as witnessed by the XRD measurements, but it seems that some small portion of the sample starts to have different optical properties. Possibly these zones can act as nucleation points for the subsequent crystallization of the whole sample. Upon heating at higher temperatures in vacuum above the crystallization temperature, Ta_2_O_5_ displays clear domains that are slightly visible in the topography but are much more pronounced in both optical signals (Fig. 5C and D). The size of the domains increases as the thermal treatment temperature increases from 1023 to 1123 K. The roughness of the sample heated just above the crystallization, i.e. at 1023 K, is 4.2 nm. Upon further heating, it decreases to 3.3 nm after the thermal treatment at 1123 K. In general, the roughness of the samples is very low; the fact that it decreases at 1023 and 1123 K can be due to the reorganization of atoms during the crystallization.

**Fig. 7:**
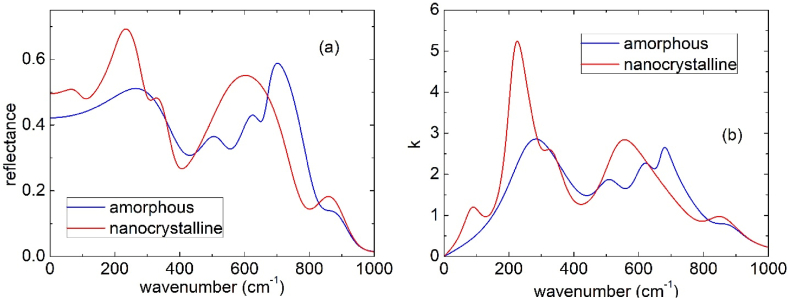
Calculated reflectance (panel a) and extinction coefficient, k, (panel b) of amorphous and nanocrystalline (randomly oriented) Ta_2_O_5_, derived from the phonon parameters reported by Bright et al. [43].

To understand the contrast appearing between different domain, we measured the near field broadband spectra of the four samples in points of apparent different brightness, as displayed in Fig. 6. For the pristine sample and the sample heated in vacuum at 988 K a maximum of the O3A signal around 730 cm^−1^ were observed (Fig. 6a and b), while for the samples heated at 1023 and 1123 K a decrease between 700 and 1000 cm^−1^ was visible (Fig. 6c and d). Above 1000 cm^−1^ an almost flat level of the O3A signal was recorded. This phenomenology is compatible with the phonon bands of Ta_2_O_5_ reported by Bright et al. [43] and Ono et al. [44]; phonons with the highest intensity are centered below 700 cm^−1^, but apparently give rise to a peak in the reflectivity of amorphous Ta_2_O_5_ around 730 cm^−1^ (Fig. 7a); this band shifts to lower wavenumbers when the material becomes nanocrystalline and a peak in the reflectivity was observed around 600 cm^−1^ (see Fig. 7a). It is interesting to note that near field IR measurements can detect the reflectance spectrum of these Ta_2_O_5_ coatings; indeed previous IR reflectance spectra collected by macroscopic normal incidence reflectivity measurements were heavily influenced by the interference fringes due to the extremely high degree of parallelism between the two surfaces of the coating [26].

Concerning the O3P near field spectra, which correspond to the absorption spectra, one can see in Fig. 6 that the pristine sample and the specimen heated in vacuum at 988 K show a large absorption band between 700 and 1000 cm^−1^ (Fig. 6a and b) with a more intense contribution centered around 800 and a weaker one around 950 cm^−1^. This phenomenology is consistent with the absorbance calculated from the data of Bright et al. [43] on amorphous tantala, which gives a peak in the absorbance centered around 875 cm^−1^ (Fig. 7b). The band around 950 cm^−1^ becomes slightly more pronounced in the sample heated at 988 K (Fig. 6c). The samples heated at 1023 and 1123 K, on the contrary, show a different absorbance spectrum (Fig. 6c and d), which moreover depends on the location chosen for the measurement: the apparent brighter domains show a decrease of the O3P signal between 700 and 1000 cm^−1^, followed by a less pronounced increase above such wavenumber; the apparent darker domains, instead, show a clear peak around 950 cm^−1^. Above the crystallization temperature it seems that at the nanoscale the samples heated in vacuum are no more homogeneous. Ono et al. [44] previously reported some reflectivity spectra of Ta_2_O_5_ in the amorphous (873 K) and crystalline state (1073 K) and observed some additional weak bands in this frequency region. However, no explanation or attribution was suggested for their presence.

**Fig. 8:**
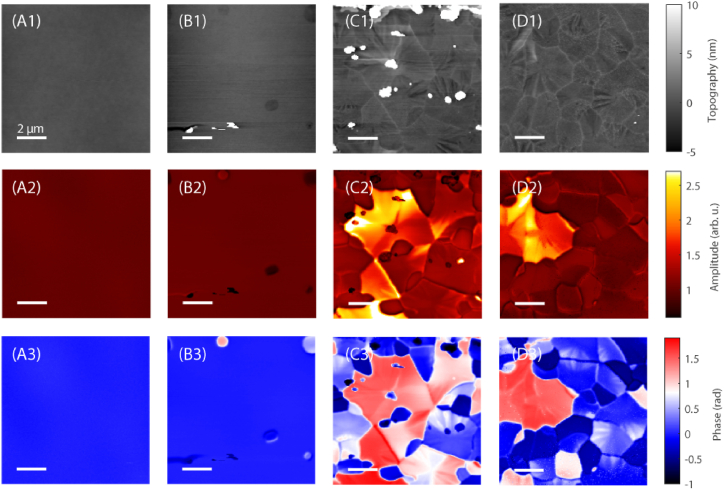
AFM topography (row 1), and near-field IR signals O3A (row 2) and O3P (row 3) at 959 cm^−1^ of the four Ta_2_O_5_ samples heated in air: at 773 K for 10 h (column A) and after fast thermal treatments at 988 K (column B), at 1023 K (column C) and at 1123 K (column D). The intensity scale span (on the right) is equal to that reported for the pristine specimen in all samples.

We speculated that due to the thermal treatment in vacuum some oxygen in a non-stoichiometric ratio with tantalum could be developed in the samples heated at the highest temperatures. Indeed, on a macroscopic scale, previous XPS measurements on similar samples suggested that oxygen was depleted within the surface layers of heated Ta_2_O_5_ [26]. For checking this hypothesis, we performed SNOM measurements on samples heated in air at 988, 1023 and 1123 K. Moreover, we added a sample heated in air at 773 K for 10 h to directly investigate the effect of the thermal treatment usually performed for the actual Virgo mirror coatings. Fig. 8 displays the AFM topography (row 1) and the near-field IR signals O3A (row 2) and O3P (row 3) measured at 959 cm^−1^ for the four Ta_2_O_5_ samples heated in air. The morphology and optical signals of the sample heated at 773 K in air (column A of Fig. 8) are similar to those of the pristine specimen (Fig. 5A), with a RMS roughness of 4.0 nm and no evidence of crystallization. After heating at 988 K in air the RMS roughness increases to 5.1 nm and there are some spots with different optical properties (column B of Fig. 8). At 1023 and 1123 K the samples display a domain structure (Fig. 8 columns C and D) analogous to that of the sample heated in vacuum (Fig. 5D). The RMS roughness of these samples are 13.2 and 6.1 nm, respectively; the reduction of the roughness at the highest temperature is certainly due to the surface flattening following the crystallization.

**Fig. 9:**
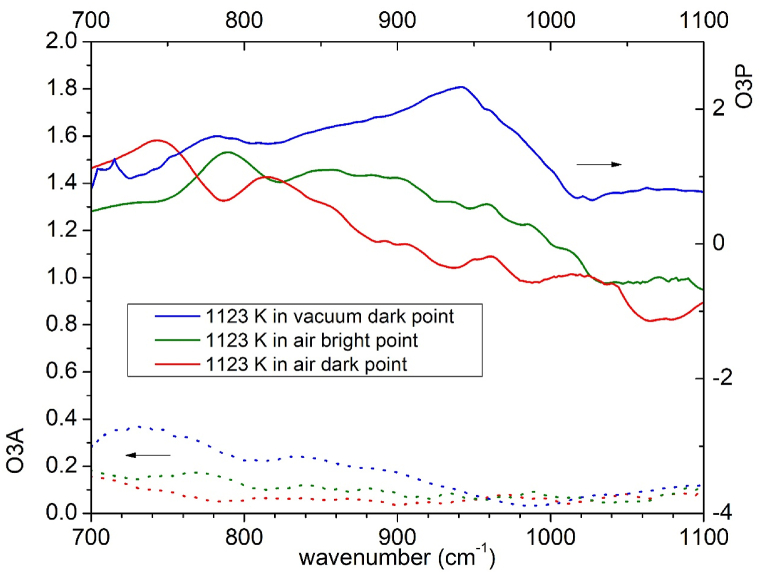
Comparison of the broadband IR absorption spectrum of Ta_2_O_5_ samples heated at 1123 K in vacuum and in air.

Fig. 9 reports the comparison of the broadband IR spectrum measured in the samples heated at 1123 K either in vacuum or in air. While in the sample thermally treated in vacuum there are single locations (corresponding to the dark spots of the O3P signal) where one can observe a clear peak around 950 cm^−1^, in no point of the sample heated in air one can find such a peak: the O3P signal decreases with increasing wavenumber as reported in Fig. 9 both for dark and bright spots. This fact further suggests that the peak at 950 cm^−1^ observed in the samples heated in vacuum is linked to a variable amount of the oxygen in these specimens.

**Fig. 10:**
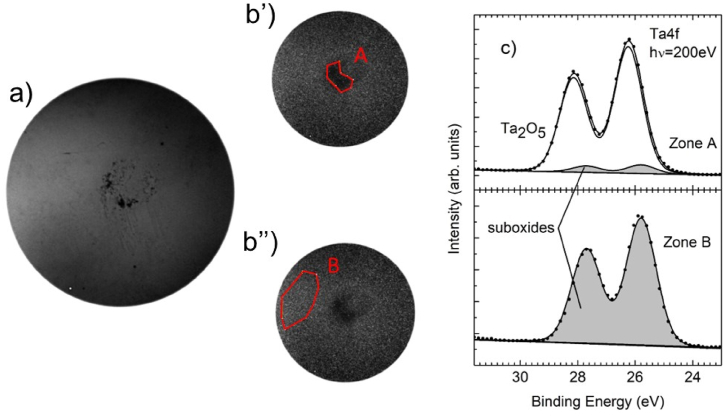
XPEEM dark field image of the sample after annealing at 1023 K in vacuum (FOV 20 μm^2^) (panel a). Panel b’) and b’’): the integrated areas chosen to extract the XPS signals. Panel c) the Ta4f core level spectra taken in the areas highlighted in panel (b), best fit line and contribution from the Ta suboxides.

To check whether an oxygen content variation on the microscopic scale was present in samples heated in vacuum, XPEEM experiments were conducted at the HERMES beamline of Soleil Synchrotron. Fig. 10a reports the dark field imaging of the XPEEM instrument on the sample heated at 1023 K in vacuum. One can note a different contrast between various regions of the sample, which could be ascribed to a local variation of the chemical environment. The Ta4f spectra were acquired in two different zones (zones A and B in panel b’ and b’’ of Fig. 10) of the sample to highlight possible changes of the local stoichiometry. The core level spectra shown in Fig. 10c were normalized to the intensity maximum and to the background towards lower binding energies. A fitting to the data was carried out using doublets with Voigt lineshape on top of a Shirley background. The branching ratio and the spin-orbit splitting (0.75 and 1.92 eV, respectively) are in agreement with the theory and the literature [45]. Apart from the main doublet attributed to the stoichiometric Ta_2_O_5_ environment (binding energy = 26.2 eV [46]), another component, shifted by about −0.45 eV, is necessary to fit to the data. In the literature, the shifted components are generally ascribed to Ta bonded to O with lower coordination numbers with respect to Ta_2_O_5_ [45,47]. The O1s spectra could not be used to corroborate this hypothesis as their lineshapes show differences below the accuracy of the technique. However, clear indications about the appearance of Ta suboxides with a spatial non-homogeneity on the micrometer length scale were obtained from the Ta4f spectra, as described. The spectra and the fitted data shown in Fig. 10c result from averaging over pixels that cover an area of several square microns. Indeed, as described in the “Materials and Methods” section, a PEEM field of view of 25 μm was utilized. If the changes occurred on a scale smaller than one micron (i.e., at the nanometer scale), the averaged spectra would not have revealed the presence of Ta suboxides. Furthermore, these measurements were conducted in various regions of the sample (not reported) to validate our observations.

The occurrence of Ta suboxides can easily explain also the appearance in the infrared spectra reported in Fig. 6 of the absorption band centered around 959 cm^−1^ in selected areas of the sample heated in vacuum at 1023 or 1123 K. Indeed, an infrared band centered between 960 and 970 cm^−1^ was previously reported for laser-evaporated TaO_2_ [48].

## 4 Conclusions

A release of 0.3 wt‰ of argon, trapped in amorphous Ta_2_O_5_ during the synthesis, is observed around 1000 K by thermal desorption spectrometry, in concomitance with crystallization, as confirmed by X-ray diffraction experiments. The smooth and flat surface of the pristine sample is preserved up to the crystallization, as witnessed by SNOM measurements; around 988 K some seeds start to appear and around 1023 K clear domains are observable, not only in topography but most of all in the optical properties of the samples. A decrease of the oxygen stoichiometry is observed by X-PEEM in samples heated in vacuum, and an additional infrared absorption band around 960 cm^−1^ is observed for oxygen deficient locations on the surface. On the contrary, no additional infrared absorbance peak is observed in samples heated in air, suggesting no significant oxygen stoichiometry change in these specimen, also at microscopic level. Further studies will be necessary in the future to understand whether the tiny concentration of argon left in amorphous tantala after a thermal treatment at 873 K could be removed by long lasting thermal treatments without reaching the crystallization temperature.

## CRediT authorship contribution statement

**A. Paolone:** Writing – original draft, Validation, Supervision, Investigation, Formal analysis, Data curation, Conceptualization. **M. Bazzan:** Investigation, Formal analysis, Data curation. **G. Favaro:** Investigation, Formal analysis, Data curation. **F. Borondics:** Investigation, Formal analysis, Data curation. **G. Nemeth:** Investigation, Formal analysis, Data curation. **F. Capitani:** Investigation, Formal analysis, Data curation. **S. Swaraj:** Investigation, Formal analysis, Data curation. **R. Belkhou:** Investigation, Formal analysis, Data curation. **R. Flammini:** Validation, Formal analysis, Data curation. **J. Teillon:** Investigation, Data curation. **C. Michel:** Investigation, Data curation. **D. Hofman:** Investigation, Data curation. **M. Granata:** Investigation, Data curation.

## Data availability statement

No data associated with this study have been deposited into a publicly available repository. The data supporting the findings of this study are available from the corresponding author upon reasonable request.

## Ethical approval

Not required.

## Funding

This research did not receive any specific funding.

## Declaration of competing interest

One of the authors, Annalisa Paolone, is an Editorial Board Member for *Heliyon* and was not involved in the editorial review or the decision to publish this article.

The other authors declare that they have no known competing financial interests or personal relationships that could have appeared to influence the work reported in this paper.

## References

[1] Adhikari R.X. (2014). Gravitational radiation detection with laser interferometry. Rev. Mod. Phys..

[2] Aspelmeyer M., Kippenberg T.J., Marquardt F. (2014). Cavity optomechanics. Rev. Mod. Phys..

[3] Matei D.G., Legero T., Häfner S.H., Grebing C., Weyrich R., Zhang W., Sonderhouse L., Robinson J.M., Ye J., Riehle F., F (2017). 1.5 μm lasers with sub-10 mHz Linewidth. Phys. Rev. Lett..

[4] Aasi J., Abbott B.P., Abbott R., Abbott T., Abernathy R.M., Ackley K., Adams C., Adams T., Addesso P., Adhikari R.X. (2015). Advanced LIGO. Class. Quantum Grav.

[5] Acernese R., Agathos M., Agatsuma K., Aisa D., Allemandou L., Allocca A., Amarni J., Astone P., Balestri G., Ballardin G., Acernese F. (2015). Advanced Virgo: a second-generation interferometric gravitational wave detector, Class. Quantum Grav.

[6] Aso Y., Michimura Y., Somiya K., Ando M., Miyakawa O., Sekiguchi T., Tatsumi D., Yamamoto H. (2013). Interferometer design of the KAGRA gravitational wave detector. Phys. Rev. D.

[7] Villar A.E., Black E.D., DeSalvo R., Libbrecht K.G., Michel C., Morgado N., Pinard L., Pinto I.M., Pierro V., Galdi V. (2010). Measurement of thermal noise in multilayer coatings with optimized layer thickness. Phys. Rev. D.

[8] Pinard L., Michel C., Sassolas B., Balzarini L., Degallaix J., Dolique V., Flaminio R., Forest D., Granata M., Lagrange B. (2017). Mirrors used in the LIGO interferometers for first detection of gravitational waves. Appl. Opt..

[9] Degallaix J., Michel C., Sassolas B., Allocca A., Cagnoli G., Balzarini L., Dolique V., Flaminio R., Forest D., Granata M. (2019). Large and extremely low loss: the unique challenges of gravitational wave mirrors. J. Opt. Soc. Am. A.

[10] Granata M., Amato A., Balzarini L., Canepa M., Degallaix J., Forest D., Dolique V., Mereni L., Michel C., Pinard L. (2020). Amorphous optical coatings of present gravitational-wave interferometers. Class. Quantum Grav..

[11] Harry G.M., Abernathy M.R., Becerra-Toledo A.E., Armandula H., Black E., Dooley K., Eichenfield M., Nwabugwu C., Villar A., Crooks D.R.M. (2007). Titania-doped tantala/silica coatings for gravitational-wave detection. Class. Quantum Grav..

[12] Durante O., Granata V., Magnozzi M., Amato A., Michel C., Pinard L., Granata M., Canepa M., Carapella G., Chiadini F., De Simone R., Fittipaldi R., Fiumara V., Pierro V., Pinto I.M., Vecchione A., Bobba F., Di Giorgio C. (2024). Role of substrate and TiO_2_ content in TiO_2_:Ta_2_O_5_ coatings for gravitational wave detectors. Class. Quantum Grav..

[13] Amato A., Magnozzi M., Shcheblanov N., Lemaître A., Cagnoli G., Granata M., Michel C., Gemme G., Pinard L., Canepa M. (2023). Enhancing titania-tantala amorphous materials as high-index layers in Bragg reflectors of gravitational-wave detectors. ACS Appl. Opt. Mater..

[14] Granata M., Amato A., Cagnoli G., Coulon M., Degallaix J., Forest D., Mereni L., Michel C., Pinard L., Sassolas B., Teillon J. (2020). Progress in the measurement and reduction of thermal noise in optical coatings for gravitational-wave detectors. Appl. Opt..

[15] Amato A., Cagnoli G., Granata M., Sassolas B., Degallaix J., Forest D., Michel C., Pinard L., Demos N., Gras S., Evans M., Di Michele A., Canepa M. (2021). Optical and mechanical properties of ion-beam-sputtered Nb_2_O_5_ and TiO_2_−Nb_2_O_5_ thin films for gravitational-wave interferometers and an improved measurement of coating thermal noise in Advanced LIGO. Phys. Rev. D.

[16] Durante O., Granata V., Neilson J., Carapella G., Chiadini F., DeSalvo R., De Simone R., Fiumara V., Pierro V., Pinto I.M., Vecchione A., Fittipaldi R., Bobba F., Di Giorgio C. (2023). Investigation of crystallization in nanolayered TiO_2_-based superlattices. Surface. Interfac..

[17] Vajente G., Yang L., Davenport A., Fazio M., Ananyeva A., Zhang L., Billingsley G., Prasai K., Markosyan A., Bassiri R., Fejer M.M., Chicoine M., Schiettekatte F., Menoni C.S. (2021). Low mechanical loss TiO_2_:GeO_2_ coatings for reduced thermal noise in gravitational wave interferometers. Phys. Rev. Lett..

[18] Song S., Cai S., Han D., García Nuñez C., Zhang G., Wallace G., Fleming L., Craig K., Reid S., Martin I.W., Rowan S., Gibson D. (2023). Tantalum oxide and silicon oxide mixture coatings deposited using microwave plasma assisted co-sputtering for optical mirror coatings in gravitational wave detectors. Appl. Opt..

[19] McGhee G.I., Spagnuolo V., Demos N., Tait S.C., Murray P.G., Chicoine M., Dabadie P., Gras S., Hough J., Iandolo G.A., Johnston R., Martinez V., Patane O., Rowan S., Schiettekatte F., Smith J.R., Terkowski L., Zhang L., Evans M., Martin I.W., Steinlechner J. (2023). Titania mixed with silica: a low thermal-noise coating material for gravitational-wave detectors. Phys. Rev. Lett..

[20] Granata M., Amato A., Bischi M., Bazzan M., Cagnoli G., Canepa M., Chicoine M., Di Michele A., Favaro G., Forest D., Guidi G.M., Maggioni G., Martelli F., Menotta M., Montani M., Piergiovanni F., Schiettekatte F. (2022). Optical and mechanical properties of ion-beam-sputtered MgF_2_ thin films for gravitational-wave interferometers. Phys. Rev. Appl..

[21] Bischi M., Amato A., Bazzan M., Cagnoli G., Canepa M., Favaro G., Forest D., Gobbi P., Granata M., Guidi G.M., Maggioni G., Martelli F., Menotta M., Montani M., Piergiovanni F., Valentini L. (2022). Characterization of ion-beam-sputtered AlF_3_ thin films for gravitational-wave interferometers. Phys. Rev. Appl..

[22] Fang S., Lu Z., Ji X., Jiao H., Cheng X., Wang Z., Zhang J. (2023). High-performance hydrogenated amorphous silicon deposited by ion-beam sputtering for gravitational-wave detectors. Phys. Rev. D.

[23] Favaro G., Bazzan M., Amato A., Arciprete F., Cesarini E., Corso A.J., De Matteis F., Dao T.H., Granata M., Honrado-Benítez C., Gutiérrez-Luna N., Larruquert J.I., Lorenzin G., Lumaca D., Maggioni G., Magnozzi M., Pelizzo M.G., Placidi E., Prosposito P., Puosi F. (2022). Measurement and simulation of mechanical and optical properties of sputtered amorphous SiC coatings. Phys. Rev. Appl..

[24] Tsai D.-S., Huang Z.-L., Chang W.-C., Chao S. (2022). Amorphous silicon nitride deposited by an NH_3_-free plasma enhanced chemical vapor deposition method for the coatings of the next generation laser interferometer gravitational waves detector, Class. Quantum Grav.

[25] Pan H.-W., Kuo L.-C., Chang L.-A., Chao S., Martin I.W., Steinlechner J., Fletcher M. (2018). Silicon nitride and silica quarter-wave stacks for low-thermal-noise mirror coatings. Phys. Rev. D.

[26] Paolone A., Placidi E., Stellino E., Betti M.G., Majorana E., Mariani C., Nucara A., Palumbo O., Postorino P., Rago I., Trequattrini F., Granata M., Teillon J., Hofman D., Michel C., Lemaitre A., Shcheblanov N., Cagnoli G., Ricci F. (2021). Effects of the annealing of amorphous Ta_2_O_5_ coatings produced by ion beam sputtering concerning the effusion of argon and the chemical composition. J. Non-Cryst. Solids.

[27] Paolone A., Placidi E., Stellino E., Betti M.G., Majorana E., Mariani C., Nucara A., Palumbo O., Postorino P., Sbroscia M. (2022). Argon and other defects in amorphous SiO_2_ coatings for gravitational-wave detectors. Coatings.

[28] Cummings R.B., Bassiri R., Martin I.W., MacLaren I. (2021). Argon bubble formation in tantalum oxide-based films for gravitational wave interferometer mirrors. Opt. Mater. Express.

[29] Bassiri R., Liou F., Abernathy M.R., Lin A.C., Kim N., Mehta A., Shyam B., Byer R.L., Gustafson E.K., Hart M., MacLaren I., Martin I.W., Route R.K., Rowan S., Stebbins J.F., Fejer M.M. (2015). Order within disorder: the atomic structure of ion-beam sputtered amorphous tantala (a-Ta_2_O_5_). Apl. Mater..

[30] Hart M.J., Bassiri R., Borisenko K.B., V′eron M., Rauch E.F., Martin I.W., Rowan S., Fejer M.M., MacLaren I. (2016). Medium range structural order in amorphous tantala spatially resolved with changes to atomic structure by thermal annealing. J. Non-Cryst. Solids.

[31] Fazio M.A., Vajente G., Ananyeva A., Markosyan A., Bassiri R., Fejer M.M., Menoni C.S. (2020). Structure and morphology of low mechanical loss TiO_2_-doped Ta_2_O_5_. Opt. Mater. Express.

[32] Martinelli A., Giovannini M., Neri M., Gemme G. (2021). Deep insights into the local structure of amorphous Ta_2_O_5_ thin films, by X-ray pair distribution function analysis. Phys. Rev. Mater..

[33] Lussier A.W., Lalande É., Chicoine M., Lévesque C., Roorda S., Baloukas B., Martinu L., Vajente G., Ananyeva A., Schiettekatte F. (2022). Hydrogen concentration and mechanical dissipation upon annealing in zirconia-doped tantala thin films for gravitational wave observatory mirrors. J. Phys.: Conf. Ser..

[34] Amato A., Terreni S., Granata M. (2020). Observation of a correlation between internal friction and Urbach energy in amorphous oxides thin films. Sci. Rep..

[35] Lalande E., Davenport A., Marchand L., Markosyan A., Martinez D., Paolone A., Rezac M., Bazzan M., Chicoine M., Colaux J.L., Coulon M., Fejer M.M., Lussier A.W., Majorana E., Martinu L., Menoni C., Michel C., Ricci F., Schiettekatte F., Shcheblanov N., Smith J.R., Teillon J., Terwagne G., Vajente G. (2024). Ar transport and blister growth kinetics in titania-doped germania-based optical coatings. Class. Quantum Grav..

[36] Belkhou R., Stanescu S., Swaraj S., Besson A., Ledoux M., Hajlaoui M., Dalle D. (2015). HERMES: a soft X-ray beamline dedicated to X-ray microscopy. J. Synchrotron Radiat..

[37] Kissinger H.E. (1956). Variation of peak temperature with heating rate in differential thermal analysis. J. Res. NBS.

[38] Palumbo O., Trequattrini F., Pal N., Hulyalkar M., Sarker S., Chandra D., Flanagan T., Dolan M., Paolone A. (2017). Hydrogen absorption properties of amorphous (Ni_0.6_Nb_0.4−y_Ta_y_)_100−x_Zr_x_ membranes. Prog. Nat. Sci..

[39] Nakamura R., Asano K., Ishimaru M., Sato K., Takahashi M., Numakura H. (2014). Stability of amorphous Ta–O nanotubes prepared by anodization: thermal and structural analyses. J. Mater. Res..

[40] Namjun K., Jonathan S.F. (2011). Structure of amorphous tantalum oxide and titania-doped tantala: 17oNMR results for sol–gel and ion-beam-sputtered materials. Chem. Mater..

[41] Xu M., Chen J., Wen Y., Du J.H., Lin Z., Peng L. (2020). ^17^O Solid-state NMR studies of Ta_2_O_5_ nanorods. ACS Omega.

[42] Nakamura R., Ishimaru M., Sato K., Tanaka K., Nakajima H., Konno T.J. (2013). Formation of highly oriented nanopores via crystallization of amorphous Nb_2_O_5_ and Ta_2_O_5_. J. Appl. Phys..

[43] Bright T.J., Watjen J.I., Zhang Z.M., Muratore C., Voevodin A.A., Koukis D.I., Tanner D.B., Arenas D.J. (2013). Infrared optical properties of amorphous and nanocrystalline Ta_2_O_5_ thin films. J. Appl. Phys..

[44] Ono H., Koyanagi K.-I. (2000). Infrared absorption peak due to Ta=O bonds in Ta_2_O_5_ thin films. Appl. Phys. Lett..

[45] Ivanov M.V., Perevalov T.V., Aliev V.S., Gritsenko V.A., Kaichev V.V. (2011). Electronic structure of δ-Ta_2_O_5_ with oxygen vacancy: ab-initio calculations and comparison with experiment. J. Appl. Phys..

[46] Atanassova E., Dimitrova T., Koprinarova J. (1995). AES and XPS study of thin RF-sputtered Ta_2_O_5_ layers. Appl. Surf. Sci..

[47] Denny Y.R., Firmansyah T., Oh S.K., Kang H.J., Yang D.-S., Heo S., Chung J.G., Lee J.C. (2016). Effect of oxygen deficiency on electronic properties and local structure of amorphous tantalum oxide thin films. Mater. Res. Bull..

[48] Wang G., Zhuang J., Zhou M. (2011). Matrix isolation infrared spectroscopic and theoretical study of the reactions of tantalum oxide molecules with methanol. J. Phys. Chem. A.

